# Volatile and Non-Volatile Allelopathic Characteristics in Thermally Processed Needles of Two Conifers

**DOI:** 10.3390/plants11081003

**Published:** 2022-04-07

**Authors:** Chan Saem Gil, Daeun Hong, Shucheng Duan, Seok Hyun Eom

**Affiliations:** Department of Smart Farm Science, College of Life Sciences, Kyung Hee University, Yongin 17104, Korea; cskil@khu.ac.kr (C.S.G.); ekdms7275@khu.ac.kr (D.H.); dsc97@khu.ac.kr (S.D.)

**Keywords:** allelopathy, bioherbicides, compost processing, coniferous volatiles, *Pinus densiflora*, *Pinus koraiensis*, thermal resistance

## Abstract

With allelopathic composts, potential merits for preventing initial weed infestations have been observed in crop transplantation. However, previous studies have rarely investigated whether high temperatures, generated during composting, decrease allelopathic ability. This study evaluated the thermal allelopathic effect of two coniferous plants (*Pinus densiflora* and *P. koraiensis*) on *Brassica napus* germination and seedling growth using their characterized allelochemical destinations. The 90 °C dry treatment of *P. densiflora* extract exhibited stronger inhibitory effect on germination than its 30 °C dry treatment. In a range from 0.25 to 1 mg mL^−1^, the germination rate was decreased to 38.1 and 64.3% of control with *P. densiflora* extract dried at 90 and 30 °C, respectively. However, *P. koraiensis* showed potent inhibition of the germination process with no statistical difference in inhibitory effects regardless of the dry temperature. Regarding *B. napus* seedling root growth, the allelopathic effects of aqueous extracts of both conifers were not reduced with the 90 °C treatment, but it was lost in seedling shoot growth. GC-MS/MS confirmed that high temperature treatment drastically decreased volatile contents to 53.2% in *P. densiflora,* resulting in reduced allelopathic abilities. However, a relatively lower decrease to 83.1% in volatiles of *P. koraiensis* accounts for less loss of the root-specific inhibitory effect on *B. napus* seedlings even after 90 °C treatment. Foliar tissues of both conifers with species-specific thermal resistance have potentially valuable functions regarding allelopathic use in horticultural compost processing ingredients, demonstrating their weed control ability during the early cultivation season where crops are transplanted in the facilitated area.

## 1. Introduction

*Pinus* (Pinaceae) is the largest genus of extant gymnosperms, widely distributed in the Northern Hemisphere [1]. Among the *Pinus* group, two conifer species, including *Pinus densiflora* Siebold and Zucc., in addition to *Pinus koraiensis* Siebold and Zucc., have been identified. Subsequently, they have been regarded as an essential forestry resource in China, Japan, and Korea. The number of needle-shaped leaves in their fascicle, two needles of *P. densiflora* and five needles of *P. koraiensis*, characterizes these two species [1].

Weed infection is a significant problem interrupting normal crop growth, potentially accounting for approximately 50% of crop yield loss [2]. Besides, weeds not only reduce crop quality but also harbor insect pests and diseases [3]. Although the use of chemical herbicides is increasing to control agricultural weeds, the indiscriminate use of herbicides results in severe ecological and environmental problems, such as soil and water contamination, occurrence of resistant weeds, weed population shifts, and dominance of minor weeds [2,4]. Therefore, current researchers are increasingly focusing on developing eco-friendly herbicides using natural products, and allelochemicals are being verified as an alternative to the use of chemical herbicides worldwide [5,6]. For example, Jabran et al. [7] reported that allelopathic plants can potentially reduce weed pressure, resulting in improved crop yields and reduced synthetic pesticide use. It has also been reported that *P. densiflora* and *P. koraiensis* are allelopathic plants, whose allelopathic effects are derived from non-volatile and volatile compounds including phenolics, flavonoids, and monoterpenes [8]. Therefore, *P. densiflora* can inhibit herbaceous invasion by releasing resin acids into the soil. Afterward, these resin acids are degraded in the soil and transformed into growth inhibitors, such as 15-hydroxy-7-oxodehydroabietate and 7-oxodehydroabietic acid [9]. Studies have further proven the allelopathic activities of *P. densiflora* and *P. koraiensis* needle tissues. As observed, aqueous methanolic extracts of *P. densiflora* needles prevented certain seedlings’ initial growth, including cress, lettuce, and alfalfa [10]. In addition, *P. koraiensis* needle water extracts inhibited the germination and growth of *Echinochloa crus-galli*, *Plantago asiatica*, *Achyranthes japonica*, *Ocnothera odorada*, and *Lactuca sativa* [11].

The application of allelopathy in cropping systems can be easily conducted by mixing the allelopathic plant residues with soil, like composts. However, this application remains limited within certain areas due to less information on allelopathic mechanisms and chemical properties. Though the organic composting materials can be encountered to substantial changes by high temperatures which are generated during the compost-producing process [12], only a few studies have investigated whether the allelopathic efficacy of plant tissue is affected by the thermal process. Furthermore, most researchers have mainly focused on compost values as nutritional suppliers to crops [13,14,15]. Instead, we tried to evaluate the allelopathic efficacy of volatile and non-volatile components in thermally processed needles. Therefore, we conducted this study to identify the allelopathic characteristics of volatile and non-volatile components in two *Pinus* needles against initial seedling growth and to determine whether thermal processes affect the allelopathic efficacy of the needles.

## 2. Results

### 2.1. Non-Volatile Assay

**Figure 1:**
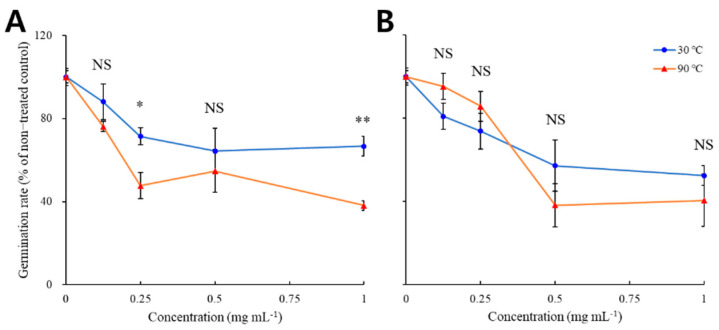
Germination patterns of *Brassica napus* seeds treated with needle extracts of *Pinus densiflora* (**A**) and *P. koraiensis* (**B**) dried at 30 or 90 °C. Asterisks indicate significant difference on Tukey’s HSD test followed by * (*p* < 0.05) and ** (*p* < 0.01). NS indicates no significant difference.

The germination rate of *Brassica napus* seeds, treated using needle extracts of *P. densiflora* and *P. koraiensis*, is shown in Figure 1. As shown, the germination rate rapidly decreased to 71 and 48% of the control with 0.25 mg mL^−1^ extract of the *P. densiflora* needle dried at 30 and 90 °C, respectively (Figure 1A). Reduction of the germination rate hardly occurred despite the concentration increase. In contrast, with *P. koraiensis*, the germination rate continually decreased to 57 and 38% of control after using 0.5 mg mL^−1^ extract of the needle dried at 30 and 90 °C, respectively.

**Figure 2:**
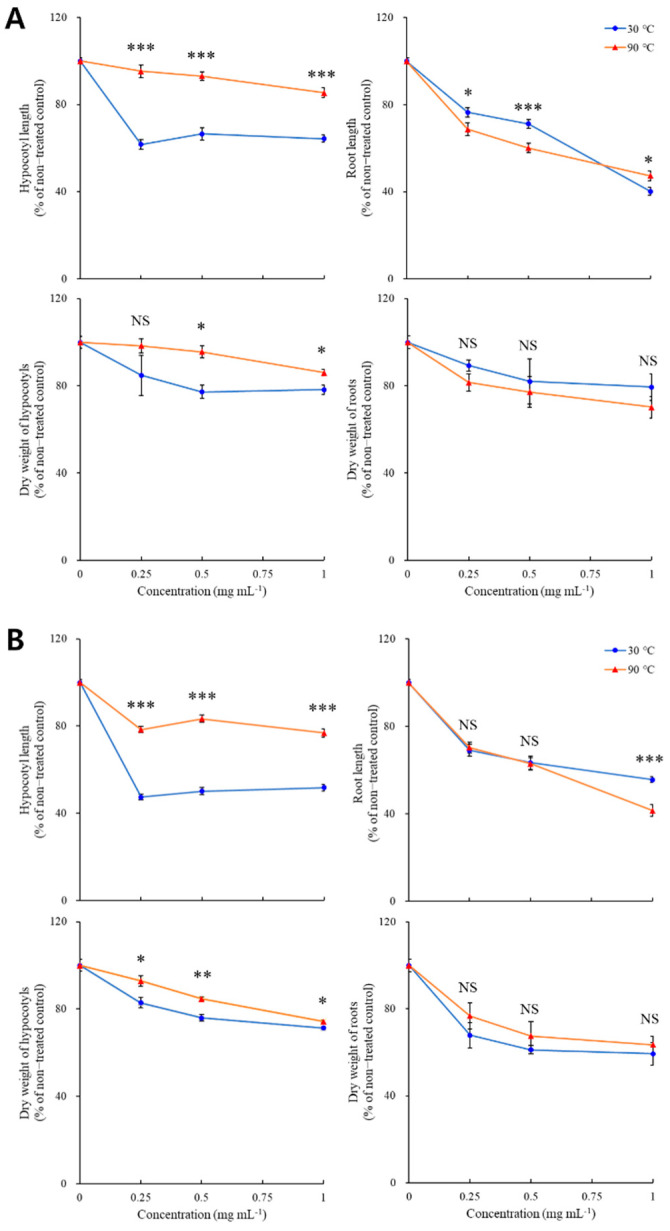
Relative growth parameters of *Brassica napus* seedlings inhibited by needle extracts of *Pinus densiflora* (**A**) and *P. koraiensis* (**B**) dried at 30 or 90 °C. Asterisks indicate significant difference on Tukey’s HSD test followed by * (*p* < 0.05), ** (*p* < 0.01), and *** (*p* < 0.001). NS indicates no significant difference.

Figure 2 shows the growth parameters of *B. napus* seedlings affected by *P. densiflora* and *P. koraiensis* needle extracts dried at 30 and 90 °C. As shown, *P. densiflora* and *P. koraiensis* needle extracts inhibited hypocotyl elongation to 62 and 48% of the control during the non-thermal treatment, using 0.25 mg mL^−1^ of the treatment (Figure 2). Furthermore, hypocotyl length was not further suppressed using treatment quantities above 0.25 mg mL^−1^. Unlike the inhibition pattern of hypocotyl growth, root growth was dose-dependently inhibited during the treatment with the two coniferous extracts. As observed with 1 mg mL^−1^ of *P. densiflora* and *P. koraiensis* extract treatment, root length was decreased to 40 and 56% of the control during non-thermal treatment, respectively (Figure 2). After the thermal process, the inhibitory effect only disappeared on hypocotyl growth. For example, when 1 mg mL^−1^ of the extract was treated, although hypocotyl elongation was inhibited to 85% in *P. densiflora* and 77% in *P. koraiensis*, root elongation was inhibited to 47 and 42% of the control in *P. densiflora* and *P. koraiensis*, respectively (Figure 2).

### 2.2. Profiles of Non-Volatiles Using HPLC and LC-MS/MS

**Figure 3:**
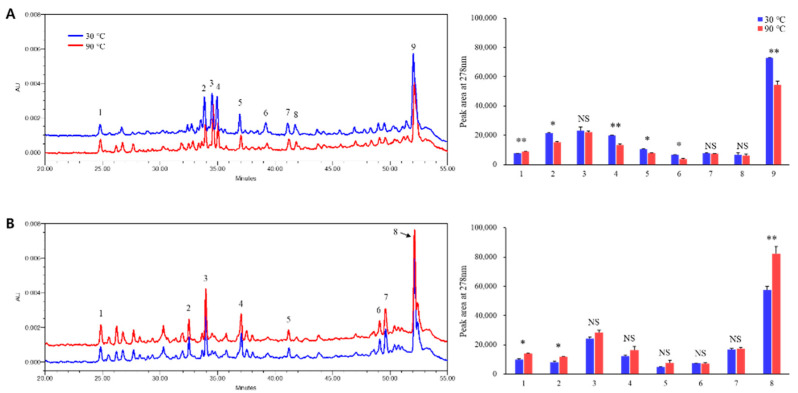
HPLC chromatograms and peak areas detected at 278 nm of separated compounds in needle extracts of *Pinus densiflora* (**A**) and *P. koraiensis* (**B**) dried at 30 or 90 °C. Asterisks indicate significant difference between dry temperatures on Tukey’s HSD test followed by * (*p* < 0.05) and ** (*p* < 0.01). NS indicates no significant difference.

High-performance liquid chromatography (HPLC) detected nine peaks in the *P. densiflora* needle extract (Figure 3A). Among the nine peaks, four components were identified using liquid chromatography coupled with tandem mass spectrometry (LC-MS/MS) (Table 1), including isoquercetrin (peak 2; *m/z* = 463.41), catechin-*O*-glucose-malonate (peak 3; *m/z* = 537.65), chrysoeriol-7-*O*-glucoside (peak 6; *m/z* = 461.49), and kaempferol 3-*O*-rutinoside-7-*O*-rhamnoside (peak 9; *m/z* = 739.57). Thermal treatment reduced most peak areas. Moreover, the 9th peak area was significantly reduced by 26% after thermal treatment at 90 °C. Alternatively, eight peaks were detected as major components in the needle extract from *P. koraiensis* (Figure 3B). Different from *P. densiflora*, after thermal treatment at 90 °C, the area of the 8th peak increased by 16%. Among these eight peaks, seven components were identified (Table 1), including schisandrin (peak 1; *m/z* = 431.91), isosteviol (peak 3; *m/z* = 363.53), lithospermic acid (peak 4; *m/z* = 537.83), quercetin-*O*-xylo-pentoside (peak 5; *m/z* = 579.70), oleuropein (peak 6; *m/z* = 539.89), tiliroside (peak 7; *m/z* = 593.75), and kaempferol 3-*O*-rutinoside-7-*O*-rhamnoside (peak 8; *m/z* = 739.57).

### 2.3. Volatile Assay

**Figure 4:**
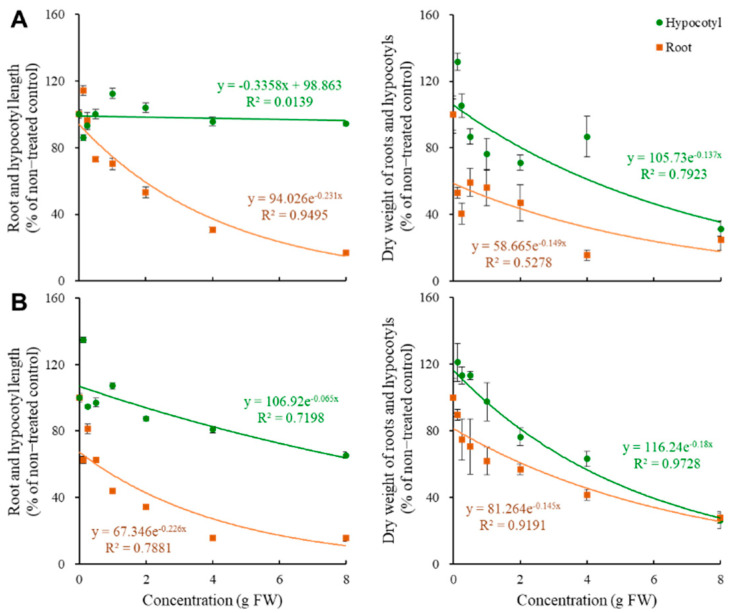
Changes of growth rates of *Brassica napus* seedlings depending on fresh weight needles of *Pinus densiflora* (**A**) and *P. koraiensis* (**B**) in volatile assay.

The allelopathic effects of volatiles from fresh *P. densiflora* and *P. koraiensis* needles on *B. napus* seedlings’ growth are shown in Figure 4. We observed that coniferous volatiles inhibited more root growth than hypocotyl growth. Furthermore, although the inhibitory effect on root growth increased with the *P. densiflora* needles’ concentration, volatiles did not affect the hypocotyl length of the *P. densiflora* needle, exhibiting 94.2% of control despite the 8 g needle treatment (Figure 4A). Alternatively, with *P. koraiensis*, both root and hypocotyl growth were dose-dependently inhibited. As observed, the dry weight of roots and hypocotyls exhibited 15.6 and 26.3% of control with 8 g fresh needle treatment, respectively (Figure 4B).

**Figure 5:**
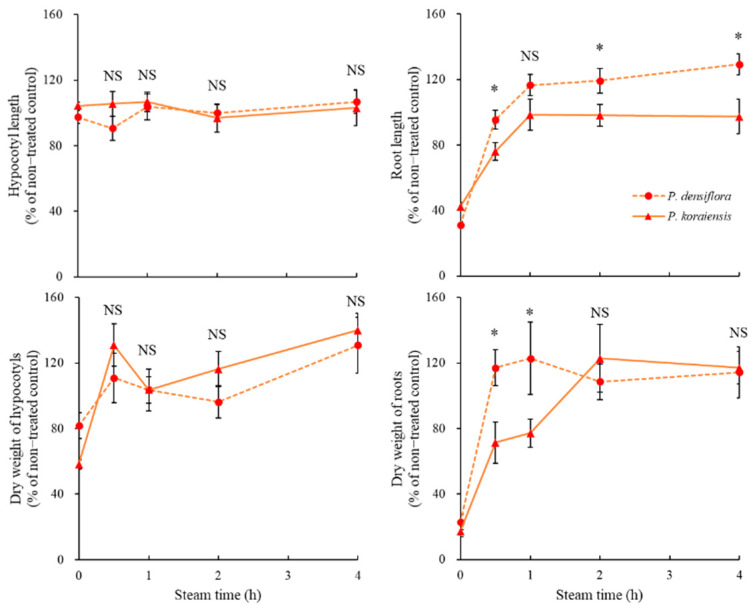
Changes of growth rates of *Brassica napus* seedlings depending on steam time of *Pinus densiflora* and *P. koraiensis* needles. Asterisks indicate significant difference on Tukey’s HSD test followed by * (*p* < 0.05). NS indicates no significant difference.

Subsequently, the allelopathic capacity of volatiles in the steamed needles of the two conifers was evaluated (Figure 5). After 4 h of steaming, the ability to inhibit hypocotyl growth was similar in both *P. densiflora* and *P. koraiensis* needles. However, the allelopathic capacity to inhibit root growth gradually decreased after one hour of steaming both coniferous needles. After more than one hour of steaming, the inhibitory power of *P. densiflora* disappeared, whereas that of *P. koraiensis* was sustained, inhibiting root length to 97.4% of the control after four hours of steamed needle treatment (Figure 5).

### 2.4. Volatile Identification and Quantification using GC-MS/MS

**Figure 6:**
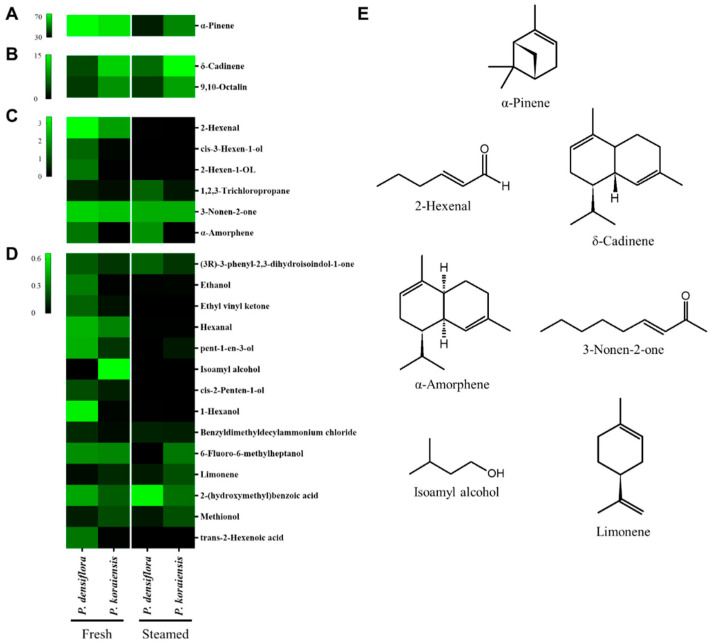
Heatmap describing relative contents of major (**A**); semi−major (**B**); semi−minor (**C**); and minor (**D**); volatiles and allelopathic volatile compounds (**E**) in either fresh or steamed needles of *Pinus densiflora* and *P. koraiensis*.

Volatile compounds were identified in *P. densiflora* and *P. koraiensis* needles, after which changes in the volatile content after 2 h of steaming were analyzed using gas chromatography tandem mass spectrometry (GC-MS/MS) (Table 2 and Figure 6). The results identified 63 and 54 volatiles in the fresh needles of *P. densiflora* and *P. koraiensis*, respectively. After steam treatment, 16 volatiles were entirely evaporated in each coniferous needle, and 7 volatiles were the same among the evaporated volatiles. Furthermore, although the number of evaporated volatiles was similar, total volatiles’ net contents differed between the two steam-processed needles. Compared to the fresh needle of each conifer, results also showed that the total volatile content decreased to 53.2 and 83.1% in steamed *P. densiflora* and *P. koraiensis* needles (Table 2 and Figure 6). Moreover, as shown in Table 2 and Figure 6, α-pinene contained the highest content in both needles, representing 75.9 and 70.7% in fresh *P. densiflora* and *P. koraiensis* needles, respectively. However, the content decreased to 36.0 and 53.9%, respectively, after steaming.

## 3. Discussion

This study established that seed germination and seedling growth are inhibited under the allelopathic effects of *P. densiflora* and *P. koraiensis* needles. Notably, their inhibitory effects on seedling growth were higher in root growth than hypocotyl growth. This difference is proposed to be because the roots directly had contact with the extracts, causing them to have a higher sensitivity to allelopathy [16,17,18]. Our study also showed that thermal treatment did not remove the allelopathic effect of the two conifer needles on root growth. Quantitative HPLC results distinctly showed the destination of each non-volatile allelochemical in coniferous extracts. As observed, most flavonoid contents were significantly decreased in *P. densiflora* needles after thermal treatment, whereas those in *P. koraiensis* needles increased. Several studies have reported a positive correlation between allelopathic efficacy and allelochemical contents [19,20,21,22]. Considering the thermal effects, the positive correlation between bioassays and HPLC results was found with the highest dose (1 mg mL^−1^) in our results. According to the other study, certain allelopathic extracts did not negatively affect seedling growth at low concentration, causing leaf extension and total biomass increase of lettuce seedlings [23]. Furthermore, it was mentioned that dose is a critical factor for exhibiting the inhibitory effects of allelochemicals in rice hull extracts [24]. Therefore, we tentatively propose that enough dose is needed to confirm the effects of allelochemicals on bioassays when extracts are applied.

Our volatile assay identified terpenoids mainly in *P. densiflora* and *P. koraiensis* needles. Results also showed that the primary compound was α-pinene, exhibiting more than 70% of the content in both conifer species. Moreover, the inhibitory effects of the volatiles on fresh foliage revealed a dose-dependent root-specific inhibition. However, unlike the extract treatment in the non-volatile assay, volatiles indirectly contacted the seedling roots. Nevertheless, constituents of essential oil and aromatic volatiles, such as monoterpenes, are easily absorbed in the soil and exhibit allelopathic effects on the rhizosphere of other plants [25,26,27]. Similarly, it has been reported that volatiles can be released from conifer needles, adsorbed onto a filter paper, and accumulated in high concentrations [26]. Another plausible explanation for root-specific inhibition is a growth characteristic difference between the root and hypocotyl. According to cellular basis, hypocotyl growth relies on elongating each cell already developed in the embryo within the seed. Besides, root growth requires proliferation and elongation of cells [28]. Yet, although volatile monoterpenes did not significantly inhibit cell expansion in both the root and hypocotyl, they inhibited cell proliferation and DNA synthesis in the root apical meristem [26].

The root-specific inhibition of the volatiles was also shown after thermal treatment. As observed, although the inhibitory effect of *P. densiflora* needles was practically eliminated through steam processes for more than one hour, that of *P. koraiensis* needles remained slight despite the increase in steaming time. However, compared to *P. koraiensis*, more volatiles were included in *P. densiflora* needles, and the total content was largely reduced through thermal treatments. After synthesis, monoterpenes are commonly stored in specialized structures, such as resin ducts, oil glands, and secretory cells in conifer needles [29]. The emission of the monoterpenes from these storage structures is related to their volatility and diffusion rate, promoted with temperature increase [30]. Additionally, volatile emission is proposed to be related to the morphological characteristics and chemical composition of storage structures. Moreover, although more resin duct quantities have been discovered in *P. densiflora* than in the *P. koraiensis* needles, they are primarily placed at the external side [1]. Therefore, compared to *P. densiflora*, *P. koraiensis* resin ducts are placed at the inner side, surrounded by the hypodermis with thickened cell walls [1]. In contrast, lower nitrogen, lignin, and cellulose contents characterize the chemical composition of the *P. densiflora* needle than the *P. koraiensis* needle [31]. These chemical properties guaranteed a relatively high resistance during thermal treatments and stability in mass reduction when *P. koraiensis* needles were decomposed [31,32]. 

## 4. Materials and Methods

### 4.1. Botanic Materials

Fresh *P. densiflora* and *P. koraiensis* needles were collected during the 2019 summer from a field at the Kyung Hee University Global Campus (N 37°14′36.0″ and E 127°04′52.6″, Yongin, Korea). *B. napus* seeds were purchased from a seed company (Budnara Co., Gwangju, Korea). 

### 4.2. Preparation of Conifer Needle Extracts

Fresh *P. densiflora* and *P. koraiensis* needles were rinsed with distilled water. After removing moisture, the needles were dried in a heat dry machine (KED-066A, C&T Co., Gwangju, Korea) at 30 °C for seven days or 90 °C for three days without light. Then, dried samples were coarsely ground using a commercial grinder and passed through a 100-mesh sieve. Subsequently, the resulting powder (10 g) from each sample was dissolved in 200 mL of 80% aqueous methanol (*v*/*v*) and agitated using a shaker (Daewonsci Inc., Bucheon, Korea) at 20 °C for 24 h. Next, the solution was filtered through qualitative filter papers (Whatman No. 2, Maidstone, UK). Finally, the solvent was removed using a vacuum rotary evaporator (Eyela, Tokyo, Japan). To remove residual solvent, the extracts were dried using a freeze dryer (IlShinBioBase Inc., Dongducheon, Korea) and maintained at 15 °C until use for non-volatile assay. 

### 4.3. Non-Volatile Assay

The germination rate and seedling growth of *B. napus* were tested to evaluate the allelopathic efficacy of non-volatiles in each *P. densiflora* and *P. koraiensis* needle extract, dried at 30 or 90 °C. To test the germination rate, we selected 20 *B. napus* seeds, excluding wrinkled and cracked seeds, followed by inoculation in a Petri dish, containing 4 mL distilled water (control) or serial concentrations (0.25, 0.5, and 1 mg mL^−1^) of each extract, diluted using distilled water. Subsequently, the dishes after seed inoculation were sealed with a parafilm, after which they were maintained in a growth chamber (Daewonsci Inc., Bucheon, Korea), completely randomized under controlled light (16 h fluorescent light per day with 50 µmol m^−2^ s^−1^), humidity (80%), and temperature (25 °C). Seed germination rate was determined by counting the number of seeds generating 1 mm or longer root daily. 

Alternatively, to test seedling growth, 20 *B. napus* seedlings generating 3 mm roots were selected and inoculated in a Petri dish containing 4 mL distilled water and serial concentrations (0.25, 0.5, and 1 mg mL^−1^) of each extract. On day five after culture, 10 similarly grown seedlings were selected among the 20 seedlings, after which their hypocotyl and root lengths were measured. Then, to measure the dry weight of the two organs, each hypocotyl and root bundle collected from the ten seedlings was entirely dried at 30 °C for 48 h. All treatments were replicated thrice.

### 4.4. HPLC Analysis

Chemical components in the needle extracts of the two conifers were analyzed using reversed-phase HPLC (Waters 2695 Alliance HPLC; Waters Inc., Milford, MA, USA), coupled with a 250 × 4.6 mm octadecylsilane column (Prontosil 120-5-C18-SH 5.0 μm; Bischoff, Leonberg, Germany). Two solvents were used as mobile phases; water with 0.1% formic acid (solvent A) and MeOH with 0.1% formic acid (solvent B). The gradient flow was as follows: 0–5% of solvent B for 0–10 min, 5–10% of solvent B for 10–20 min, 10–20% of solvent B for 20–30 min, 20–40% of solvent B for 30–50 min, and 40–70% of solvent B for 50–62 min. Furthermore, the flow rate of the mobile phases was 1.0 mL min^−1^, and the sample injection volume was 5 μL. Then, resulting peaks were monitored at 278 nm using a Waters 996 photodiode array detector.

### 4.5. LC-MS/MS Analysis

Molecular weights of the flavonoids in the two coniferous extracts were determined using an LC-MS/MS with a Thermo-Finnigan LTQ-Orbitrap instrument (Thermo Fisher Scientific, Waltham, MA, USA) at NICEM in Seoul National University (Seoul, Korea). Then, data acquisition was conducted with Xcalibur^TM^ software (ver. 4.3., Thermo Fisher Scientific Inc., MA, USA). In contrast, MS and MS/MS were operated through an electrospray ionization source in the negative ion mode, recorded in the range of 150 to 2000 *m/z* and 50 to 2000 *m/z*, respectively. Besides, nitrogen gas was used as the sheath gas, whose flow rate was kept at 10 L min^−1^. The capillary temperature was also maintained at 300 °C, nebulizer pressure was set at 45 psi, whereas fragmentation and capillary voltages were 0.2 kV and 4.5 kV, respectively. Additionally, the collision energy was set at 35 Ev, after which individual compounds were identified by comparing mass data and λ_max_ results to previously reported values.

### 4.6. Volatile Assay 

The volatile assay was conducted using a method described in a previous study [33], with some modifications. First, fresh or steam-processed needles were coarsely mashed using a mortar with simultaneous liquid nitrogen pouring. Then, the fresh needles were, respectively, weighed (0.125, 0.25, 0.5, 1, 2, 4, and 8 g) and packaged using one layer of cheesecloth. For the steaming process, fresh needles were placed in boiling water for 0.5, 1, 2, and 4 h, after which 4 g of the steam-processed needles were weighed and packaged with one layer of cheesecloth. Next, each sample package was positioned above 10 cm from the bottom of a glass bottle (500 mL), after which 20 *B. napus* seedlings, generating 3 mm root, were inoculated on a moistened filter paper (Whatman No.3, Maidstone, UK) placed in the glass bottle. The bottles were tightly sealed with caps and wrapped in a parafilm to avoid any leak of volatiles. Finally, the seedlings were cultured in a growth chamber (Daewonsci Inc., Bucheon, Korea) under the same conditions as the non-volatile assay. On day five after culture, seedling growth was evaluated using the same method as the non-volatile assay.

### 4.7. GC-MS/MS Analysis

Fresh or two-hour steamed needles were ground using a mortar with simultaneous liquid nitrogen pouring, after which 1.5 g ground sample and 1 g NaCl were added to the ground samples in a solid-phase microextraction (SPME) amber vial containing 6 uL 1,2,3-trichloropropane. Subsequently, an SPME system (Xcalibur^TM^ ver. 4.3, Thermo Fisher Scientific Ind., Waltham, MA, USA) was used to isolate the volatile compounds using a fiber coated with a 65 μm polydimethylsiloxane/divinylbenzene film layer (fused silica 24 Ga). Then, analysis was conducted using a GC (Trace1310, Thermo Fisher Scientific Inc., Waltham, MA, USA), equipped with a triple quadrupole mass spectrometer (TSQ8000, Thermo Fisher Scientific Inc., Waltham, MA, USA) and a DB-Wax column (60 m × 0.25 mm, 0.50 μm, Agilent Technologies, Santa Clara, CA, USA). Additionally, a helium carrier gas was used at a flow rate of 2 mL min^−1^ and 230 °C inlet temperature. Next, we maintained the ramp temperature in the GC oven at 40 °C for 5 min, increased the temperature to 120 °C for 8 °C min^−1^, then to 160 °C for 2 °C min^−1^, and 240 °C for 4 °C min^−1^. The sample was finally held at the final temperature for another 10 min. Afterward, volatile compounds were identified using the NIST/EPA/NIH Mass Spectral Library (ver. 2.0), then the volatiles’ quantification was represented as each compound’s relative peak area (%).

### 4.8. Statistical Analysis

Statistical analyses were conducted using the SAS Enterprise Guide (ver. 4.3, SAS Institute Inc., Cary, NC, USA). Then, significant differences among the treatments in these experiments were evaluated using Tukey’s studentized range test at *p* < 0.05.

## 5. Conclusions

The allelopathic capacity of two *Pinus* species showed root-specific inhibition, revealed using dose-dependent needle extracts. In the coniferous needle extracts, flavonoid glycosides were identified as non-volatile allelochemicals. A positive correlation between bioassays and chemical contents was revealed in the application of high doses of the extracts. In addition, from the volatile assay results, seedlings were root-specifically suppressed through allelopathic volatiles in the two coniferous needles. Moreover, both coniferous needles contained large terpenoid and α-pinene quantities. However, after the thermal process, the decrease in volatile contents was lesser in *P. koraiensis* than in *P. densiflora*. It has been proposed that keeping the allelopathic capacity from thermal treatment is attributed to the chemical and morphological properties of *P. koraiensis* needles. Considering the allelopathic characteristics of the needles on germination and initial root growth, the application of allelopathic compost is considered more beneficial for a cropping system. In addition, it is proposed that coniferous needles have potential as eco-friendly herbicides to control initial weed growth due to their thermal stability and root-specific inhibition.

## Figures and Tables

**Table 1:** Metabolites identified in needle extracts of *Pinus densiflora* and *P. koraiensis* by LC-MS/MS analysis.

Species	No.	RT (min)	[M−H]^−^	MS/MS	Identification
*P. densiflora*	1	26.40	−	−	NI ^1^
	2	34.00	463.41	179.0/271.0/301.1/343.1/417.4/445.2	Isoquercetrin
	3	34.33	537.65	299.3/327.1/328.8/469.1/490.9/515.8	Catechin-*O*-glucose-malonate
	4	34.50	509.54	163.0/179.0/311.1/367.3/385.2/473.3/491.2	NI
	5	35.84	551.88	327.2/329.2/341.1/358.8/359.5/491.1	NI
	6	40.70	461.49	139.1/165.1/193.1/243.1/29.0/298.1/299.0/341.0/342.1/433.2	Chrysoeriol-7-*O*-glucoside
	7	41.40	493.82	315.2/316.4/447.2/448.3	NI
	8	42.70	493.68	259.1/315.2/426.0/447.2/447.9	NI
	9	48.80	739.57	229.0/285.1/286.1/289.1/435.2/453.2/454.2/575.3/593.3/620.3/885.5	Kaempferol 3-*O*-rutinoside-7-*O*-rhamnoside
*P. koraiensis*	1	26.30	431.91	153.0/223.0/307.3/343.1/385.1/386.2/399.3	Schisandrin
	2	29.60	571.37	316.1/375.3/467.3/525.4/541.3	NI
	3	30.67	363.53	135.0/147.0/165.0/179.1/201.2/221.1/239.0/273.3/315.1/345.1/346.2	Isosteviol
	4	34.30	537.83	163.1/207.4/299.1/327.3/329.2/345.0/477.3/491.1/519.5	Lithospermic acid
	5	43.93	579.70	178.9/255.1/301.1/343.0/433.0/434.2/489.2/561.2	Quercetin-*O*-xylopentoside
	6	46.11	539.89	207.0/331.3/372.9/432.9/492.9	Oleuropein
	7	46.40	593.75	178.9/203.0/285.1/293.1/299.0/300.1/316.2/417.2/447.3/547.1/576.4	Tiliroside
	8	48.80	739.70	229.1/285.0/286.1/289.0/429.1/453.1/454.3/575.1/593.2/594.3/638.9/680.3	Kaempferol 3-*O*-rutinoside-7-*O*-rhamnoside

^1^ NI: not identified.

**Table 2:** Profiles and relative contents of volatile compounds in either fresh or steamed needles of *Pinus densiflora* and *P. koraiensis* by GC−MS/MS analysis.

Compound	RT	*m/z*	*P. densiflora*		*P. koraiensis*	
			Fresh	Steamed	Fresh	Steamed
(3R)-3-phenyl-2,3-dihydroisoindol-1-one	6.52	209.24	0.24 ± 0.02	0.25 ± 0.01	0.14 ± 0.00	0.14 ± 0.00
Ethanol	9.18	46.07	0.32 ± 0.01	0.02 ± 0.00	ND ^1^	ND
3,3,5-Trimethylheptane	11.04	142.28	0.11 ± 0.00	0.01 ± 0.00	ND	ND
α-Pinene	11.48	136.23	75.91 ± 0.82	35.95 ± 0.33	70.69 ± 0.10	53.90 ± 0.00
Ethyl vinyl ketone	11.56	84.12	0.25 ± 0.00	0	ND	ND
Hexanal	13.09	100.16	0.46 ± 0.03	0.01 ± 0.00	0.34 ± 0.01	0.01 ± 0.00
pent-1-en-3-ol	14.69	86.13	0.45 ± 0.00	0	0.14 ± 0.00	0.07 ± 0.00
2-Hexenal	16.39	98.14	3.37 ± 0.09	0.01 ± 0.00	2.08 ± 0.06	0
Isoamyl alcohol	17.98	88.15	ND	ND	0.66 ± 0.00	0
cis-2-Penten-1-ol	18.55	86.13	0.20 ± 0.00	0	ND	ND
1-Hexanol	19.41	102.17	0.62 ± 0.05	0	ND	ND
cis-3-Hexen-1-ol	20.46	100.16	1.34 ± 0.12	0	0.19 ± 0.00	0
2-Hexen-1-OL	21.07	100.16	1.54 ± 0.02	0.01 ± 0.00	ND	ND
1,2,3-Trichloropropane	23.16	147.43	0.48 ± 0.01	1.30 ± 0.03	0.27 ± 0.00	0.36 ± 0.00
Benzyldimethyldecylammonium chloride	26.19	311.90	0.11 ± 0.00	0.10 ± 0.00	ND	ND
6-Fluoro-6-methylheptanol	29.10	148.22	0.36 ± 0.00	0	0.34 ± 0.00	0.30 ± 0.00
3-Nonen-2-one	29.11	140.22	2.72 ± 0.01	2.25 ± 0.02	2.55 ± 0.01	2.25 ± 0.00
Limonene	30.45	136.24	0.05 ± 0.00	0.08 ± 0.00	0.11 ± 0.00	0.21 ± 0.00
α-Amorphene	32.81	204.35	1.52 ± 0.01	1.89 ± 0.01	ND	ND
δ-Cadinene	35.87	204.35	4.47 ± 0.02	6.40 ± 0.06	12.35 ± 0.13	15.29 ± 0.00
9,10-Octalin	36.10	136.23	3.55 ± 0.08	3.52 ± 0.02	8.69 ± 0.10	9.50 ± 0.00
2-(hydroxymethyl)benzoic acid	37.10	152.15	0.42 ± 0.01	0.63 ± 0.02	0.24 ± 0.00	0.29 ± 0.00
Methionol	37.46	106.19	ND	ND	0.19 ± 0.00	0.21 ± 0.00
trans-2-Hexenoic acid	43.90	114.14	0.30 ± 0.04	0	ND	ND
Trace compounds (below than 0.1%)			1.21 ± 0.00	0.77 ± 0.01	1.02 ± 0.00	0.56 ± 0.00
Total			100.0	53.2	100.0	83.1

^1^ ND: not detected.

## Data Availability

All data included in the main text.
